# Investments for Professional Men

**Published:** 1913-05

**Authors:** John W. Van Allen

**Affiliations:** Buffalo


### Investments For Professional Men

BY JOHN W. VAN ALLEN, ESQ.
Buffalo

FOR some reason or other, which I believe is quite unknown to you, it has been deemed quite proper to consider the members of your profession as active buyers of mining, promotion and other alluring stocks, calculated to sufficiently remunerate the seller, but in most instances leaving the patient in a more or less exhausted state financially or otherwise.

### Life Insurance.

The necessary education of a professional man today is fraught with so much of expense that upon the beginning of his career he is likely to have incurred obligations, the payment of which ought, in some manner, to be assured in the event of his sudden demise. To this end a carefully selected life insurance policy covering the amount of his obligations is probably the least expensive—the premiums being payable in installments—of any form of guarantee against loss.

While not generally practised, I am an earnest advocate of the insuring of one's life to his estate in a sum equal at all times to the amount of any obligations by notes, mortgages, contracts or otherwise outstanding at any time.

When, during the course of your professional life, you have been able to capture and to accumulate a family, their well-being, in case of your death, should be safeguarded by further insurance, even though you may have among your properties stocks, bonds or other securities which would be valuable assets in case of death. The reason for this is more apparent when you consider that estates cannot be closed ordinarily within a period of one year and during that time stocks, bonds or securities might have to be sold at a sacrifice in order to provide actual cash for living expenses to the members of your family, so suddenly deprived of the cash producer. The life insurance in this case provides practically immediate cash available to your wife or family, and it should be payable direct to them, with which to pay expenses until an opportunity has arrived either to sell your securities at good advantage or to readjust themselves to the changed conditions brought about by your death.

### Savings Banks.

Under the laws of the State of New York, and, in fact, under the laws of practically all of the states, savings banks are permitted to invest only in the most conservative securities. When you deposit your money in these institutions you receive, at least in this city, interest thereon at the rate of 4% per annum. The institution takes your funds and invests them for you in conservative securities, generally receiving an income greater than 4%, the difference in the amount paid you as interest and the amount received from the security as interest representing the cost of operating the bank. In other words, the bank makes your investments for you, charging as its commission or services the difference between what you get and what it gets.

To a man unacquainted with securities in general, and to a limited amount, it is generally wiser to pay savings bank experts this commission than to endeavor to make the selections of securities himself.

### Bonds.

An income greater than sufficient to pay living and business expenses, insurance premiums and a savings bank deposit may, with judicious handling, be invested in approved stocks and bonds, yielding a much higher interest income than savings bank interest. If, for instance, after making a careful study of the securities which savings banks purchase, you are able to select good securities which those banks would purchase for themselves, you will be able to save the amount which you would pay for their services, thus securing, instead of 4%, probably between 4 and 5%, and if by mortgages on real estate, perhaps 6% annually.

There are good bonds which yield a fair rate of interest and have a general market where they can be readily sold, which do not come strictly under the head of savings bank investments, although they are practically as safe; and careful inquiries among investment bankers will usually disclose such bonds for sale.

### Stocks.

Stocks at the best are more or less speculative, dependent largely upon the character of the business in which the company issuing them is engaged, and to an equally great or greater extent upon the management and control of the business. In order to make them fairly conservative, there should be in the company, in addition to assets equal to all of the current debts, mortgage and bond obligations, sufficient assets to constitute a fair proportion of the par value of the stock, and an earning capacity in all of the assets together, taken as a unit, to meet all charges and yield a fair dividend to the stockholders.

Of course, preferred stock is more safeguarded than common stock generally, both as to distribution of principal in case of dissolution and as to the payment of regular dividends before any dividends are paid upon the common stock. It must be remembered, however, that preferred stocks usually carry only limited dividends, while common stock ordinarily pays dividends equal to the net earnings after paying expenses and other obligations, including the stated dividends on the preferred stock, and the amount of these, of course, depends again upon the earning capacity of the assets as a unit and the character of the management.

### CONCLUSIONS.

I believe, that based upon a modest income as a start and gradually increasing as the years go by, the accumulated income of professional men should be invested about as follows:

1. Life insurance payable to your estate, equal at all times to the amount of your financial obligations.

2. Life insurance payable to your family sufficient to provide them with ready cash in case of death, and for a sufficiently long time thereafter to readjust themselves to new conditions and realize to the most advantage on other securities which you have accumulated during your lifetime.

3. A moderate savings bank account.

4. Approved bonds with a wide market, yielding a fair income.

5. Preferred stocks issued against assets at least double the amount of the par value of the amount of such stock issued, in a company whose annual net profits are equal or greater than twice the amount of the required preferred stock dividends.

6. Common stocks with a fairly wide market in companies whose earning capacity for a series of years, and the payment of dividends during that time, warrants the belief of safety of principal and continuity of dividend payments.

7. When you have a fair amount of property represented in the foregoing investments, then certain not too highly speculative transactions may be indulged in with that surplus, your situation being such at the time that a good sized estate will not be prejudiced by the losses which you make in endeavoring to make large profits by taking long chances.

NOTE.—The Cave Men Association, Inc., of New York, was incorporated last fall to "expose fraud in the exploitation of worthless mining properties and to standardize mining practice."

